# Effectiveness of Biologic Factors in Shoulder Disorders

**DOI:** 10.2174/1874325001711010163

**Published:** 2017-02-28

**Authors:** Dimitrios Giotis, Ashkan Aryaei, Theofanis Vasilakakos, Nikolaos K. Paschos

**Affiliations:** 1 Department of Trauma & Orthopaedic Surgery, University of Ioannina, Ioannina, Greece; 2 Department of Biomedical Engineering, University of California, Davis, USA

**Keywords:** Biologic factors, Effectiveness, Growth factors, Healing, Osteoarthritis, Platelet rich-plasma, Rotator cuff, Stem cells

## Abstract

**Background:**

Shoulder pathology can cause significant pain, discomfort, and loss of function that all interfere with activities of daily living and may lead to poor quality of life. Primary osteoarthritis and rotator cuff diseases with its sequalae are the main culprits. Management of shoulder disorders using biological factors gained an increasing interest over the last years. This interest reveals the need of effective treatments for shoulder degenerative disorders, and highlights the importance of a comprehensive and detailed understanding of the rapidly increasing knowledge in the field.

**Methods:**

This study will describe most of the available biology-based strategies that have been recently developed, focusing on their effectiveness in animal and clinical studies.

**Results:**

Data from *in vitro* work will also be briefly presented; in order to further elucidate newly acquired knowledge regarding mechanisms of tissue degeneration and repair that would probably drive translational work in the next decade. The role of platelet rich-plasma, growth factors, stem cells and other alternative treatments will be described in an evidence-based approach, in an attempt to provide guidelines for their clinical application. Finally, certain challenges that biologic treatments face today will be described as an initiative for future strategies.

**Conclusion:**

The application of different growth factors and mesenchymal stem cells appears as promising approaches for enhancing biologic repair. However, data from clinical studies are still limited, and future studies need to improve understanding of the repair process in cellular and molecular level and evaluate the effectiveness of biologic factors in the management of shoulder disorders.

## INTRODUCTION

Shoulder joint disorders cause chronic pain and functional disability influencing adversely the quality of life. Particularly, shoulder pain is considered as one of the three most common causes of musculoskeletal complaints [1]. Most commonly reported shoulder disorders include traumatic and degenerate tears of the rotator cuff (RC) (RC tendinopathy), subacromial impingement syndrome and bursitis, frozen shoulder, bicipital tendonitis, and glenohumeral/acromioclavicular arthritis [2].

Among them, two major disorders are identified; RC tendinopathy and osteoarthritis (OA). RC tendon pathology is reported as the source of approximately 30-70% of shoulder pain disorders [1, 3, 4]. Interestingly, more than 50% of the patients older than 60 years-old demonstrate pathologies, such as RC impingement and partial- or full-thickness tear [5]. Shoulder OA is another common disorder that causes disability and pain, with an increasing incidence after the age of 50 years old [6, 7]. Over the last years, an increasing incidence of shoulder disorders is reported, indicating more comprehensive diagnosis and improved understanding in the related pathology and treatment [6-8]. The significance of shoulder disorders becomes clear when considering the emerge of new treatment approaches and the increased burden of shoulder pain that results in higher cost of treatment over the last decades [3, 4, 9-11].

The etiopathogenesis of shoulder disorders is far more complex than in load bearing joints, due to the fact that shoulder joint withstands a wide range of compressive, shear, and tensile loads [12, 13]. RC pathology has been attributed to intrinsic or extrinsic theories. The first involves the theories of hypoperfusion, degeneration, microtrauma and apoptosis, while the second refers to chronic impingement syndrome, overuse and other multifactorial causes of progressive degeneration [14-20]. Regardless the etiologies, shoulder diseases concerning especially the RC, alter progressively the shape of acromion, causing further narrowing of the subacromial space. Furthermore, upward migration of the humeral head after rotator cuff deficiency also contributes in inflammation, pain, and potential functional limitation [1, 4, 21, 22]. Similarly, for glenohumeral cartilaginous degeneration, several factors have been considered risk factors, such as trauma, (impact and joint dislocation), osteonecrosis, inflammatory disorders, and infection [23-26].

Management of shoulder disorders includes conservative treatment, which involves analgesics, anti-inflammatory drugs, local anesthetics, steroid injections and physiotherapy, and, when conservative treatment fails, open or arthroscopic surgery for more advanced disorders. Debridement of the degenerative tissue or repair of the rotator cuff tear with re-attachment of the ruptured tendon to the bone with non-absorbable sutures, may offer significant pain relief. When osteoarthritis develops, the only viable treatment option is joint reconstruction. However, both conservative and operative treatment may have limitations, be ineffective or lead to repair failure. For instance, in cases of large massive tears with extreme degeneration and poor tissue quality, the rate of healing failure is considerably high, estimated at up to 75% [27-32]. Therefore, further improvement of treatment success in shoulder disorders is a critical aspect of patient care.

**Fig. (1):**
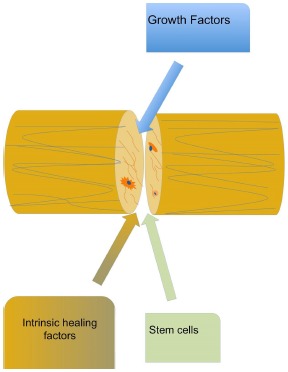
Main biologic factors that are associated with tendon healing process.

Several risk factors for tendon repair failure have been identified, both patient-related and management related. For example, patient`s age and smoking are among the most important factors for re-tear [33-36]. Further, insufficient suture strength, size, and chronicity of tear are also implicated in tissue healing [32, 36-38]. Tendon advanced degeneration, as it occurs in late tears, is characterized by reduced cell numbers, poor matrix organization, and poor vascularization, thus, preventing full capacity of tissue healing [39-42]. On the other hand, the occurrence of failures in shoulder disorders repair even in younger age might imply the presence of conditions in the healing process associated with biologic factors that are expressed early in the repair phase (Fig. **1**) [39, 43]. Since large chronic RC tears have high failure rates certain factors exist in those cases that are implicated in the failure of rotator cuff repairs [27]. It has become apparent that the rate-limiting step is the inability of the healed tendon to mimic the structural and functional characteristics of native enthesis, the normal tendon-to-bone transition zone [44, 45]. Instead of a four-zone structure rich in type I collagen, the healed tendon-to-bone interface is characterized by a layer of fibrocartilage rich in collagen type III [44, 45]. It is the quality of tendon-bone healing that appears to be most probably responsible for the high failure rate of rotator cuff repair.

Several new techniques present promising data that could potentially change the treatment approach to shoulder pathology as known today [10, 23]. In that aspect, great concern has been expressed towards improving the biological environment and tissue’s healing capacity [46-52]. The usage of biologic factors can be proven as an essential key for more effective management of shoulder disorders at an earlier stage [46-52]. Biologics can be used either as part of conservative management or as adjuvants to surgical therapy.

**Fig. (2):**
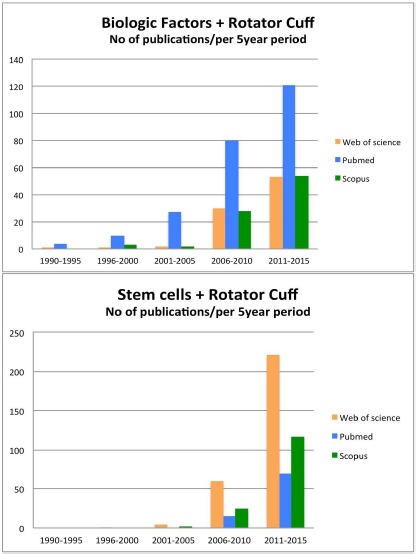
**(a).** Number of studies published regarding biological factors and rotator cuff per 5-year period in Web of Science, PubMed, and Scopus databases. **(b).** Number of studies published regarding stem cells and rotator cuff per 5-year period in Web of Science, PubMed, and Scopus databases.

The interest in biologic repair in shoulder has increased enormously over the last 5 years. As shown in Fig. (**2**), the number of studies published about “biologic factors” and “rotator cuff” (used as the most representative and popular condition for shoulder specific pathology) has rapidly increased over the last 5 years (Fig. **2a**). Similar exponential trend in the published studies was also observed when the search in PubMed, Web of Science, and Scopus databases included the terms “stem cells” and “rotator cuff” (Fig. **2b**). This interest is indicative for the need of alternative effective treatments for shoulder disorders, and highlights the need of a comprehensive and detailed knowledge of the rapidly increasing amount of the relevant literature.

The purpose of the review is to summarize the numerous biology-based strategies that have been proposed for both non-operative and surgical management of shoulder joint disorders *in vitro*, but most importantly in animal and clinical studies. We will focus on the effect of biological factors used in shoulder disorders, describing the role of platelet-rich plasma (PRP), and growth factor use. Furthermore, the findings of application of mesenchymal stem cells (MSC), matrix metalloproteinases (MMP) inhibitors, scaffolds, and gene therapy will be described. The emphasis will be on rotator cuff pathology, since the applications of biologic therapies for early shoulder osteoarthritis is limited, and mostly concern other joints, such as the knee.

## PLATELET RICH PLASMA (PRP)

Platelet-rich plasma (PRP) gained popularity for shoulder disorders after introduced in the last decade as a biological component that could potentially improve rotator cuff tendinopathy [53, 54]. PRP is a fraction of whole blood with supra-physiological concentration of platelets, that, once activated, releases various growth factors, inflammatory cells, and proteins, that subsequently enhance stromal and mesenchymal stem cell proliferation and prevent fibrous scar tissue healing [41, 55, 56]. The preparation process includes separation of platelets from whole blood mainly through centrifugation, and a mixing step where agents that activate platelets are added [57-59]. The initial release of growth factors from alpha granules activates additional differentiation and secretion of growth factors after 7-10 days, which coincides with the inflammatory and repair phases of tendon [57, 60].

There is a wide variability among the different techniques for PRP preparation, which reflects the wide variability of the effectiveness of the prepared PRPs among the different studies [61]. The main growth factors that are released from platelets are transforming growth factor-beta (TGFb), platelet-derived growth factor (PDGF), vascular endothelial growth factor (VEGF), hepatocyte growth factor, epithelial growth factor, and insulin-like growth factor 1 (IGF-1) [57]. These autologous growth factors are regarded as beneficial and accelerative for healing through cell proliferation, collagen regeneration, and revascularization [62-64]. Interestingly, the existence or not of these factors varies depending on the applied commercial system (*e.g.* number of available platelets, presence of anticoagulants and activators) with those containing higher leukocyte levels to be considered as more effective [65].

**Fig. (3):**
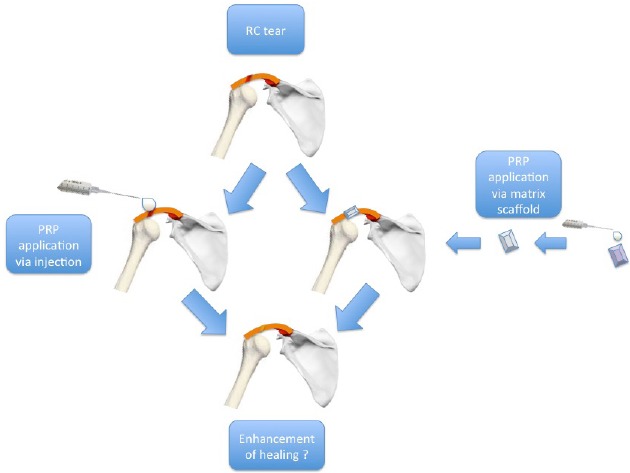
Treatment strategies for biologic factor clinical application. An example of PRP use.

PRP can be applied over the repaired tissue, either directly with injection or through matrix scaffold [57] (Fig. **3**). In 2009, four categories of PRPs were defined based on the presence of leukocytes and fibrin architecture: a) Pure Platelet-Rich Plasma (P-PRP) – or Leukocyte-Poor Platelet-Rich Plasma (without leukocytes and with a low density fibrin network after activation). b) Leukocyte-and Platelet-Rich Plasma (L-PRP) (with leukocytes and a low-density fibrin network after activation). c) Pure Platelet-Rich Fibrin (P-PRF) – or Leukocyte-Poor Platelet-Rich Fibrin (without leukocytes and with a high-density fibrin network). d) Leukocyte-and Platelet-Rich Fibrin (L-PRF) (with leukocytes and a high-density fibrin network) [66, 67].

Matras in 1970 was the first who investigated the effect of growth factors released from PRP in skin wound healing of rats [68]. However, it was only the last decade that further investigation on the effects of different forms of PRP (*in vitro* or *in vivo*) on shoulder disorders was implemented. PRP injections for rotator cuff tendinopathy were shown to have promoting effects on tendon proliferation, collagen gene expression, and angiogenesis [41, 69]. Recently, these properties of PRP were confirmed *in vitro* demonstrating that growth factors released from PRP may improve cell proliferation of human tenocytes from degenerative rotator cuff and promote synthesis of extracellular matrix [70, 71].

Several *in vivo* animal studies were recently performed examining the effect of PRP on shoulder diseases. Enhancement of tendon-bone healing after local administration of autologous PRP in rabbits with chronic rotator cuff tears was evident, as assessed by histological and biomechanical testing [72]. Similar histological and biomechanical data with improved tendon-bone healing were reported after the application of intra-articular autologous PRP in rats during surgical repair after acute rotator cuff tear [73]. Another animal study showed enhancement of tendon-to-bone interface healing with PRP application regardless of the mode of application, *i.e.* injection *versus* sponge carrier [74].

Despite the theoretic basis and the promising results from *in vitro* and *in vivo* studies, there is a great controversy in the clinical practice for the effectiveness of different types of PRP on shoulder cuff disorders. In a meta-analysis, it was demonstrated that there was no clear clinical benefit or effect on the overall re-tear rate from the use of PRP after arthroscopic rotator cuff repair, especially for massive tears [75]. For medium to small tears it seems that there is a tendency of lower re-tear rate [75]. In particular, a prospective randomized double blind controlled level one study, included 88 patients with small to medium sized cuff lesions where the tears were repaired with a double row procedure [76]. No statistically significant differences were observed with the use of PRP at 16 months postoperatively, concerning both clinical and imaging parameters [76]. Another prospective randomized double-blind clinical trial demonstrated that there are only short-term (at 3 months) benefits regarding the clinical outcome (constant score, simple shoulder test and subjective shoulder value) and MRI, from the use of L-PRP injection during arthroscopic rotator cuff repair [77]. However, after 2 years follow-up, no statistical differences were stated concerning these parameters and only pain was lower in the L-PRP as compared to the control group [77]. In a prospective cohort study, it was observed that despite the fact that PRP gel application to arthroscopic rotator cuff repairs did not accelerate recovery in terms of pain, range of motion, strength, functional scores, or overall satisfaction, magnetic resonance imaging demonstrated a lower re-tear rate in the PRP group in comparison to the control group, but again, this did not reach a statistically significant difference [78]. Similar results were also demonstrated for PRP on at risk for failure rotator cuff repair, where the use of PRP increased the re-tear rate [79]. However, it cannot be ignored that in 2 out of 16 cases, there was a local infection [79].

More recently, a randomized controlled trial involving 60 patients, investigated the effect of postoperative, repeated, ultrasound-guided injections of PRP to the repaired supraspinatus tendon after double-row arthroscopic repair [80]. They found that there was no enhancement in early tendon-bone healing, functional recovery, range of motion, strength or pain [80]. A level one prospective randomized double-blind study that examined two groups of 27 patients each operated for cuff tear repair (PRP group *versus* control group) confirmed the above results [81]. PRP did not promote better clinical results at 24-month follow-up and there were no differences regarding the re-tear rate between the two examined groups [81]. The ineffectiveness of PRP to provide satisfactory results in arthroscopic cuff repair was also supported by another level one randomized, double-blind, controlled clinical trial of that found that the use of PRP in arthroscopic repair of rotator cuff tears was not beneficial for cuff healing with no functional (clinical assessments) or structural (arthro - magnetic imaging resonance (MRI)) improvements observed one year postoperatively [82]. In fact, in a prospective randomized level two study, platelet-rich plasma fibrin matrix (PRPFM) not only failed to show a positive effect on clinical outcome, but also may have a negative effect by altering the biological interface between tendon and bone [83].

On the other hand, a level three study that investigated the influence of PRPFM augmentation in two groups of 20 patients operated of a cuff repair with and without PRPFM, found a higher re-tear rate in the control group (without PRPFM) as assessed by MRI [84]. In addition, a level one prospective randomized study which included patients operated with an arthroscopic single-row technique for large rotator cuff tear, reported better repair integrity in the PRP group (with platelet-leukocyte membrane) as compared to the control one (without platelet-leukocyte membrane) [85]. Application of PRP improved significantly structural outcomes for large to massive rotator cuff tears, as evidenced by a decreased re-tear rate, and improved shoulder function after 1-year follow-up [86]. It was also demonstrated that the use of autologous L-PRP was related with a lower re-tear rate in patients undergoing arthroscopic repair of large or massive rotator cuff tears, based on postoperative MRI evaluation [87]. However there was no correlation with the functional outcome 2 years postoperatively [87].

Regarding the role of PRP without a concurrent operative treatment of chronic rotator cuff tendinopathy, it was found that PRP injections were no more effective in improving quality of life, pain, disability, and shoulder range of motion than placebo, in patients with chronic tears who were treated with an exercise program [88]. Opposing results were found when an ultrasound-guided PRP injection was compared with dry needling in rotator cuff disease [89]. PRP led to a progressive reduction in pain and disability at six months after treatment in comparison to control [89]. Encouraging results for the potential of PRP to heal the muscle-tendon unit of the cuff at the level of degenerative tissue in refractory rotator cuff tendinopathy was also supported by data that intra-lesional injection of PRP resulted in improved functional and MRI outcome [90].

Platelet related growth factors has been shown *in vitro* to enhance chondrocyte proliferation, proteoglycan synthesis, collagen synthesis, and promotes chondrogenesis [91-94]. Despite this knowledge, that there are no clinical studies reporting outcomes for shoulder osteoarthritis. Recently, a technique for the treatment of focal chondral lesions at the glenohumeral joint with a combination of micronized allogeneic cartilage and platelet-rich plasma was described [95]. An increasing number of animal and clinical studies showed promising findings of the use of PRP in the knee and other joints [96-100]. Regarding other shoulder disorders, an interesting clinical report demonstrated that several cytokines are involved in the pathophysiology of synovial hyperplasia and capsular fibrosis detected in patient with adhesive capsulitis [101]. Additional research would provide better understanding of the exact mechanism of action of biological factors in the glenohumeral disorders, which is necessary to support whether PRP has any role in shoulder osteoarthritis treatment.

In summary, it is difficult to reach safe conclusions regarding the benefits from using of PRP in management of rotator cuff disorders due to the conflicting clinical evidence. Despite the encouraging *in vitro* results, the outcomes from clinical trials did not confirm the beneficial effect of PRP. The different surgical techniques, the different rehabilitation protocols, and most importantly, the different types of PRP seem to play an important role in outcome. Detailed research is important to clarify composition, effective dosage, and mode of action for PRPs [102]. Additional randomized multidisciplinary clinical trials are also needed to determine the effectiveness and clarify whether PRP application in rotator cuff tears and other glenohumeral disorders is beneficial or not.

## GROWTH FACTORS

Apart from the growth factors that are released from PRP and have been discussed previously, several growth factors have been evaluated independently for their potential contribution to shoulder disorders healing. In general, growth factors are signal molecules that participate in the events of cell proliferation, protein synthesis, and as modulators of various phases of inflammation [103, 104]. They can be produced from various types of cells, such as inflammatory cells, platelets, and fibroblasts and their effectiveness depends on binding to specific receptors [103]. Due to their anabolic role, various growth factors are released to promote cellular proliferation and matrix deposition in early stages of tendon healing [43, 105]. Interestingly, growth factors initially appear at the proximal site of the myotendinous insertion and significantly earlier than at the distal site [43]. An absence at an early stage may be associated with underperfusion of the critical zone [43].

The main growth factors associated with shoulder disorders are the following: a) basic fibroblast growth factor (bFGF), b) platelet derived growth factor (PDGF), c) insulin-like growth factor-1 (IGF-1) d) transforming growth factor-beta (TGF-β), e) vascular endothelial growth factor (VEGF) and f) bone morphogenetic proteins 2, 7, 12, 13, 14 (BMP-2,7,12,13,14) [27, 88, 106]. bFGF is considered decisive in the proliferation and remodeling phases as potential stimulator of angiogenesis and fibroblast proliferation [43, 102, 106, 107]. PDGF plays an important role in early stages of healing process by stimulating and regulating the synthesis of IGF-1 and TGF-β [43, 102, 103, 106, 108]. IGF-1 and TGF-β are both released in the early phase of healing and they stimulate proliferation and migration of fibroblasts and other local cells [43, 106-108]. They also have a key role in chondrocyte proliferation and homeostasis [109, 110]. VEGF acts as a stimulator of angiogenesis of epi- and intratendinous vessels being present during inflammation, proliferation, and remodeling phase of healing process. In addition, it stimulates the secretion of bFGF [103, 107, 108]. BMPs are multifunctional cytokines, which are members of the transforming growth factor-β superfamily. They are regarded as factors that induce signal transduction for stem cell differentiation into osteoblastic cell [111, 112].

### In Vitro

Several *in vitro* studies were performed to analyze the effect of these biologic factors on shoulder disorders, inspired by their participation in the tendon-bone healing process. bFGF was demonstrated having a stimulatory effect on the proliferation of rotator cuff tendon cells in a dose-dependent way, while simultaneously it suppressed secretion of collagen type I and type III [113]. Genetical modification of tenocytes with VEGF gene demonstrated very limited effects on the promotion of collagen production in an *in vitro* model [114]. BMP-2 and BMP-7 have shown in an *in vitro* model to increase collagen type I production and expression on tenocytes-like cells [115]. In parallel it was observed that BMP-7 increased cell activity [115]. A combination of bone marrow stromal cells (BMSCs) and growth and differentiation factor-5 (GDF-5) was shown to promote tendon healing, while treatment using either growth factor alone was unsuccessful. Administration of GDF-5 in muscle-derived stem cell implantation in an *in vitro* tendon healing appeared to improve the outcome of tendon repair [116]. Furthermore, sustained release of granulocyte-colony stimulating factor (G-CSF) ensured *via* the use of vesicular phospholipid gels (VPGs) showed some promising findings *in vitro* [117]. More recent data, identified gene expression as a new potential direction, as demonstrated by an *in vitro* study that identified sry-type homeobox protein-9 (SOX-9) and regulator of G-protein signaling-10 (RGS-10) as targets for gene therapy [118].

### In Vivo

#### bFGF

An *in vivo* canine model revealed that bFGF accelerated the cell-proliferation phase of tendon healing, while simultaneously it promoted neovascularization and inflammation in the earliest phases following the suturing of the tendon [119]. However there was no enhancement in mechanical and functional properties of the repair [119]. Moreover, two different *in vivo* rat studies from a single group [102, 103] found that local application of bFGF accelerated the tendon-bone remodeling process of rotator cuff tendon defects reconstructed with acellular dermal matrix grafts [120, 121].

#### PDGF

In an *in vivo* sheep study, recombinant human PDGF-BB FiberWire (Arthrex, North Naples, FL, USA) when used to coat sutures that were used for rotator cuff tendon-bone repair, demonstrated enhanced healing histologically, but ultimate load to failure was no different to standard suture repairs [122]. In an analogous study an interpositional graft consisting of recombinant human PDGF-BB and a type I collagen matrix was implanted in an ovine model of rotator cuff repair [123]. After 12 weeks, it was found that this combination of PDGF-BB with a type I collagen matrix had the potential to augment surgical repair of rotator cuff tears [123]. Restoration of normal crimp patterning and collagen-bundle alignment in a rat rotator cuff repair model after delivery of cells expressing PDGF-BB on a polyglycolic acid scaffold was also suggested [124]. A significant increase in collagen and DNA synthesis in PDGF-b induced tendon fibroblasts was demonstrated in the same model [47].

#### IGF-1

Regarding IGF-1, using fibroblasts from rat tendons that were cultured and transferred with the gene of IGF-1, found that maximum load to failure and toughness were improved in the tendon repairs [47]. In an *in vivo* model, IGF-1 reached a peak expression in the early phase of supraspinatus tendon healing, earlier compared to bFGF and PDGF, indicating its involvement at the early phase of tendon healing [43].

#### TGF-β

Concerning the growth factor TGF-β, delivery of TGF-β3 with a heparin/fibrin-based TGF-β3 delivery system to the tendon-to-bone insertion in a rat model accelerated the healing process of supraspinatus tendon with enhanced vascularity, cellularity, inflammation, and cell proliferation at early stages [125]. Considerable improvements were also observed in structural properties at 28 days and in material properties at 56 days as compared to the controls [125]. Another relevant *in vivo* animal study investigated the delivery to the tendon-to-bone interface of repaired rat supraspinatus tendons of TGF-β3 in an injectable calcium-phosphate (Ca-P) matrix [126]. It was observed that the augmentation with the osteoconductive Ca-P matrix at the tendon-bone repair site was associated with new bone formation, increased fibrocartilage, improved collagen organization, and increased collagen type I/type III ratio at the healing tendon-to-bone interface in the early postoperative period after rotator cuff repair [126]. Plus, the addition of TGF-β3 significantly improved strength of the repair at 4 weeks postoperatively, which implied more mature healing [126]. On the contrary, TGF-β1 was suggested that over-activates inflammatory response and lead to scar formation [127]. In an animal flexor tendon repair study, the use of neutralizing antibodies against TGF-β1 could reverse this effect [127]. Examining the exact role of TGF-β1 and TGF-β3 at the tendon-to-bone insertion of repaired rat supraspinatus tendons by using an osmotic pump delivery system revealed that TGF-β1 increased type III collagen production, which was consistent with a scar-mediated healing response of low quality, but at the same time TGF-β3 application did not show improvement in healing [128].

#### VEGF

VEGF is among the growth factors contained in PRP, and therefore certain aspect of its actions has been described earlier. Solely, VEGF was demonstrated to be expressed in the early phase after tendon injury [129]. Due to its role in angiogenesis, it was proposed that it plays a critical role in tendon healing [130]. Recently, an *in vivo* model of rotator cuff healing showed that VEGF improved biomechanical properties of repaired tissue [131]. However, in two clinical studies, VEGF expression was associated with motion pain and synovial proliferation in patients with impingement syndrome, while a correlation with diabetes was also reported [132, 133].

#### BMPs

With reference to BMPs, supraspinatus tendon repairs treated with BMP-2 in a rat model were significantly stronger than the controlled group and histological analysis showed more organized healing after 4 and 6 weeks [51]. Similar results were presented when the effect of injectable hydrogel with BMP-2 on rotator cuff repair in rabbits was examined [134]. BMP-2 provided a powerful inductive ability between the tendon and the bone and enhanced tendon-bone healing through the neo-formation of fibrocartilage [134]. Beneficial results were also reported in a sheep animal study where delivery of recombinant human BMP-12 in a collagen or hyaluronan sponge resulted in accelerated healing of acute full-thickness rotator cuff repairs [135]. Contrarily, the application of mesenchymal stem cells genetically modified to over-express BMP-13 in a supraspinatus tendon-bone repair did not enhance healing, which suggests that further research is required in order to clarify the effect of BMP family [136].

#### Combination of Growth Factors

Apart from the research directly related to PRP, to our knowledge, there is only one *in vivo* study in the literature that investigated the effect of combining several different growth factors in shoulder disorders [137]. In this study, several growth factors from cortical bone extract (BMP-2 to BMP-7, TGF-β1, TGF-β2 TGF-β3 and FGF) were implanted with a type I collagen sponge into a healing supraspinatus repair of 72 sheep [137]. It was observed that the combination of growth factors resulted in formation of new bone, fibrocartilage, and soft tissue, with a parallel improvement of biomechanical properties at the repair site. Interestingly, further analysis implied that scar tissue formation might have occurred rather than tissue regeneration [137]. Considering the contradictory outcomes also seen with PRP application, which represents a mixture of different growth factors, as well as the complexity in growth factor interactions, it is recognizable that the different growth factors may have interrelating, synergistic, or sometimes antagonistic roles in tissue repair [43, 109, 137]. The exact mechanism of action of growth factors in musculoskeletal tissues needs to be further explored, as its exploration will serve as the basis for additional clinical applications.

## STEM CELLS

In the last decade, the use of mesenchymal stem cells (MSCs) to enhance rotator cuff tendon healing and to manage musculoskeletal shoulder disorders has gain ground, due to their potential to differentiate towards different target cells and their anti-inflammatory and angiogenic characteristics [104, 138]. Stem cells, either embryonic or adult stems cells, are undifferentiated cells that under the influence of endogenous and exogenous factors have the potential to differentiate into certain adult cell types of mesenchymal origin (*i.e.*, bone, fat, tendon, muscle, cartilage) with various applications in tissue engineering and orthopedic surgery [139-142]. According to the tissue sources, the main types of adult MSCs are: a) Bone marrow-derived MSCs (BM-MSCs), b) Tenocyte-derived MSCs (T-MSCs), c) Adipose-derived MSCs (A-MSCs), d) Muscle-derived MSCs (M-MSCs). Recently, additional sources of MSCs have been identified such as the synovia, periosteum, dermis, and peripheral blood, thus, further increasing the potential of future clinical applications [104, 141, 143].

Especially for rotator cuff healing, the main source of MSCs is the bone marrow, which can be easily accessed through aspiration even from the iliac crest, or from the proximal humerus [138-140]. These BM-MSCs after cultivation have the potential under suitable stimulation (*e.g.* insulin) to differentiate to tendon cells [138, 139, 144]. T-MSCs can be isolated from the supraspinatus or from the biceps tendon and are considered of extreme interest for rotator cuff repair enhancement [145, 146]. A-MSCs might be easy to be obtained, but their ability to differentiate is obviously reduced as compared to BM-MSCs [147]. In terms of other potential sources of MSCs, synovial cells were recently isolated from the subacromial bursa and glenohumeral joint, and they are considered as a good source of MSCs with a high potential for applications [148, 149].

Despite the apparent similar biological potential of different types of MSCs, several *in vivo* animal and clinical studies have explored the effect of different forms of MSCs on shoulder cuff disorders. In all the executed animal studies, MSCs were applied directly to the healing site or were delivered on an appropriate carrier matrix, which functioned as a scaffold as it can be realized below. In the first reported case control animal study, in 80 rats that were operated for supraspinatus tendon repair BM-MSCs isolated from rats` long bones were injected on the repair side. Despite their presence at the repair site, BM-MSCs were not proven to improve structural biomechanical aspects of healing [48]. In an attempt to address this inefficiency, additional differentiation factors may need to be combined with BM-MSCs therapy, attributing the inability to improve healing to poor signaling that did not induce differentiation of transplanted cells [48]. Indeed, in a subsequent series of studies, a) genetic modification of MSCs to over-express the developmental gene membrane type 1 matrix metalloproteinase (MT1-MMP), increased both structural and material biomechanical properties and increased the amount of fibrocartilage at the tendon-to-bone interface [150], b) MSCs genetically modified with scleraxis, a transcription factor believed to direct the tendinous attachments to bone, improved histological cartilage formation at the insertion site, with analogous enhancement of biomechanical properties [151], c) contrarily, MSCs genetically modified to over-express BMP-13 was not successful to enhance healing in a rat model of rotator cuff repair [136]. Thus, further enhancement of MSC differentiation seems to be critical, while a multi-factorial approach is more likely to induce appropriate healing and regeneration [136, 150, 151].

In an animal model, additional drilling to the greater tuberosity during the rotator cuff repair process was suggested that allowed BM-MSCs to migrate into the suture zone, and to infiltrate the site of rotator cuff repair, contributing to early postoperative rotator cuff healing [152]. Furthermore, a polyglycolic acid sheet scaffold with seeded BM-MSCs enhanced the expression of type I collagen and increased the mechanical strength of a regenerated rotator cuff tendon in a rabbit model [153]. Favorable results regarding the survival of MSCs were shown when these cells were encapsulated and implanted in open-cell polylactic acid scaffold in acute rabbit rotator cuff defect [154].

Regarding the other types of stem cells, local administration of A-MSCs indicated their potential to improve muscle function and tendon healing and decrease fatty infiltration after cuff repair [155]. In addition, the injection of highly purified muscle derived cells (MDCs) into the supraspinatus tendon of rats resulted in the engraftment of transplanted cells with morphology similar to resident tendon fibers [156]. However it was unclear whether MDCs had the capability to improve rotator cuff healing [156]. Finally, tendon stem/progenitor cells (TSPCs) that were isolated from human fetal Achilles tendon, cultured, and subsequently implanted in a rabbit rotator cuff defect, not only enhanced tendon regeneration by differentiating into tenocytes, but also prevent immunological rejection by secreting anti-inflammatory cytokines [157].

Despite the extensive research in animal experimental studies in reference to the role of MSCs in shoulder cuff healing, with rather promising results, there are only few clinical studies that evaluate the safety of clinical application of stem cells in rotator cuff tears. In a clinical study, 14 patients with complete rotator cuff tears were repaired with sutures and the repair was augmented with mononuclear stem cells from iliac crest bone marrow [158]. After one-year follow-up, in all patients tears showed integrity according to MRI criteria and 13 out of 14 patients had improved clinical outcome [158]. Recently, long term results of bone marrow-derived MSCs used as an adjunct to single-row rotator cuff repair at the time of arthroscopy were reported [159]. At 10 year follow up, re-tear of rotator cuff was found in only 13% of the MSC-treated group, *versus* 56% re-tear rate of the control group, indicating enhancement of healing from MSCs and durability of the clinical outcome [159]. Finally, two separate case reports that describe the treatment of rotator cuff tear with the use of a dermal allograft with the addition of MSCs and PRP also report favored outcome [160, 161].

In conclusion, the literature in respect to the effect of stem cells on shoulder disorders and their ability to facilitate regeneration of normal tendon-to-bone insertion and restrain growth of scar tissue is limited to almost only animal studies. Promising data are seen recently from the relevant clinical studies. However, further basic research is required in order to better understand differentiation potential and the relationship between MSCs with other biologic and signaling factors. Additional clinical research should be performed with randomized controlled trials to verify the encouraging results of experimental studies on humans.

## OTHER FACTORS

The role of matrix metalloproteinases (MMPs) and their natural inhibitors known as tissue inhibitors of metalloproteinase (TIMPs) as potential biologic tools in rotator cuff tendon-bone healing is also under investigation over the last years. MMPs have the ability to shape connective tissue and to degrade collagen and elastin of the extracellular matrix (ECM), which is substantial for healing process of tendons [162] Imbalance in the equilibrium between MMPs and TIMPs in rotator cuff tendons, results in elevated levels of MMPs which can lead to degenerative rotator cuff tissue and cause tendon tears [162]. Further, it has been found that there was an increase in expression of MMP-1, MMP-2, and MMP-3 in rotator cuff tears, which appeared to influence the healing process [163-167]. Doxycycline was used to inhibit MMP-3 activity during rotator cuff healing in an animal study; the authors observed that inhibition of MMP led to improved biomechanical quality of the scar tissue as regards to increased load to failure, and better collagen fiber organization, which may offer a novel biological pathway to augment tendon-bone healing [168].

The use of scaffolds to mechanically stabilize the repair site has also been an area of active research. Natural and synthetic extracellular matrix (ECM) scaffolds have been used in order to share load as well as to provide a conductive chemical and/or structural environment for repair healing and remodeling [169-171]. Synthetic scaffolds have the advantage of being acellular and therefore do not induce a tissue response. Natural ECM needs intensive processing so as to remove cells and cell remnants that may cause intense tissue reactions. There are numerous questions related to their indication, surgical application, safety, mechanism of action, and efficacy that remain to be clarified or addressed, and other alternatives *via* scaffold-less approach may be worth exploring [172, 173].

Other biologic factors that might offer promising results in acceleration tendon-bone healing are the human growth hormone and proteoglycans [174, 175]. Nevertheless, there is still a long way for the scientific community to investigate the effectiveness of these factors in shoulder tendinopathy by animal studies and clinical trials. Finally, new horizons have opened up with the development of gene therapy and transgenic therapy, as described above in some examples. However the safety of these biological approaches for human trials needs to be addressed in future studies [104].

## CONCLUSION

Increased morbidity of shoulder disorders led to an increased interest in the potential effect of biologic factors to improve cuff tendon healing or prevent cartilage degeneration. Despite the promising results of PRP in animal studies regarding augmentation of cuff tendon due to activation and release of growth factors, clinical trials have not confirmed their beneficial effect. Due to the continued challenges in clinical practice, the application of different growth factors and mesenchymal stem cells appears as promising alternatives for enhancing biologic repair. Despite promising outcomes reported in both *in vitro* and *in vivo* studies, data from clinical studies are still limited. Thus, future well-designed basic science studies are needed to improve understanding of the repair process in cellular and molecular level, but also, appropriately designed clinical trials are of paramount importance to elucidate the effectiveness of biologic factors in the management of shoulder disorders.

## LIST OF ABBREVIATIONS

A-MSCs: = Adipose-derived Mesenchymal Stem Cells.
bFGF: = basic Fibroblast Growth Factor.
BM-MSCs: = Bone Marrow-Derived Mesenchymal Stem Cells.
BMP: = Bone Morphogenetic Proteins.
BMSCs: = Bone Marrow Stromal Cells.
Ca-P: = Calcium-Phosphate.
ECM: = Elastin of the Extracellular Matrix.
G-CSF: = Granulocyte-Colony Stimulating Factor.
GDF-5: = Growth and Differentiation Factor-5.
IGF-1: = Insulin-like Growth Factor 1.
L-PRF: = Leukocyte-and Platelet-Rich Fibrin.
L-PRP: = Leukocyte-and Platelet-Rich Plasma.
MDCs: = Muscle Derived Cells.
MMP: = Matrix MetalloProteinases.
M-MSCs: = Muscle-derived Mesenchymal Stem Cells.
MSC: = Mesenchymal Stem Cells.
MT1-MMP: = Membrane Type 1 Matrix Metalloproteinase.
OA: = Osteoarthritis.
PDGF: = Platelet-Derived Growth Factor.
P-PRF: = Pure Platelet-Rich Fibrin.
P-PRP: = Pure Platelet-Rich Plasma.
PRP: = Platelet-Rich Plasma.
PRPFM: = Platelet-Rich Plasma Fibrin Matrix.
RC: = Rotator Cuff.
RGS-10: = Regulator of G-protein Signaling-10.
SOX-9: = Sry-Type Homeobox Protein-9.
TGFb: = Transforming Growth Factor-beta.
TIMPs: = Tissue Inhibitors of Metalloproteinase.
T-MSCs: = Tenocyte-derived Mesenchymal Stem Cells.
TSPCs: = Tendon Stem/Progenitor Cells.
VEGF: = Vascular Endothelial Growth Factor.
VPGs: = Vesicular Phospholipid Gels.
